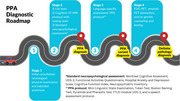# Optimizing primary progressive aphasia diagnosis: The development of a localized framework

**DOI:** 10.1002/alz70857_102825

**Published:** 2025-12-25

**Authors:** Florentina Morello Garcia, Loana De Los Santos, Carolina Agata Ardohain Cristalli II, Maria Eugenia Tabernero, Vanina Banjsak, Ismael Luis Calandri, Lucia Crivelli, Ricardo Allegri, Nahuel Magrath Guimet

**Affiliations:** ^1^ Institute of Neurosciences (INEU), Fleni‐CONICET, Buenos Aires, Buenos Aires, Argentina; ^2^ Fleni, Buenos Aires, Buenos Aires, Argentina; ^3^ Fleni, Buenos Aires, Argentina; ^4^ GBHI, San Francisco, CA, USA

## Abstract

**Background:**

Diagnosing primary progressive aphasia (PPA) is challenging. It requires establishing language impairment as predominant and classifying the PPA variant based on speech and language features. Neuroimaging and biomarker assessments are recommended for greater diagnostic accuracy. However, difficulties in diagnosis are common, particularly in low‐ and middle‐income countries (LMICs), where socio‐economic variables exacerbate these challenges. This work presents the PPA diagnostic roadmap developed at Fleni (Argentina) to address these barriers.

**Method:**

The PPA diagnostic roadmap combines extensive clinical experience with insights from existing literature, integrating clinical, neuropsychological, neuroimaging, and biomarker data to identify and classify PPA variants. Based on the diagnostic criteria by Gorno‐Tempini et al. (2011), the framework has been adapted to the local population to streamline diagnostic processes and reduce delays in resource‐limited settings.

**Result:**

Patients undergo an initial assessment by a neurologist, including a neurological‐physical examination and extended anamnesis (Stage 1, see Figure 1). During the same visit, Stage 2 tests are ordered. These include laboratory tests (e.g., blood count, thyroid profile, vitamin B12), a cognitive 3T MRI protocol with resting‐state, and a standard neuropsychological assessment (MoCA, UDS‐3, and socioemotional and functional scales). Stage 2 results enable the neurologist to establish a baseline PPA diagnosis, identifying primary progressive language impairment and obtaining imaging‐based confirmation. Determining the specific PPA variant, however, requires a language‐specific assessment (Stage 3). The PPA protocol at Fleni evaluates key aspects of language and speech to determine the patient's linguistic profile. It includes the MLSE, Token Test, Boston Naming Test, Pyramids and Pharaohs Test, FTLD‐module of UDS‐3, and a speech protocol. Additionally, FDG‐PET, amyloid‐PET biomarkers, and/or genetic counseling and testing are requested (Stage 4).

**Conclusion:**

Stages 1 and 2 of the diagnostic roadmap constitute possible scenarios considering the region's capabilities. Laboratory tests and MRI are viable, and the standard neuropsychological assessment includes readily available tests in Spanish. However, Stages 3 and, especially, Stage 4 are currently achievable only in specific socio‐economic contexts due to limiting factors (e.g., limited access to language specialists, high costs of specialized studies, lack of healthcare coverage). This roadmap serves as a framework to guide professionals in the region and optimize the diagnostic process.